# Trajectories of SARS-CoV-2 viral shedding among admitted patients at a tertiary-care center in California, 2020–2022

**DOI:** 10.1017/ash.2023.329

**Published:** 2023-09-29

**Authors:** Ralph Tayyar, Melanie Kiener, Jessica Ferguson, Jane W. Liang, Gustavo Contreras Anez, Guillermo Rodriguez Nava, Caitlin A. Contag, Alex Zimmet, Krithika Srinivasan, John Shepard, Benjamin A. Pinsky, Lucy Tompkins, Aruna Subramanian, Jorge Salinas

## Abstract

**Background:** SARS-CoV-2 viral load decreases over time after illness onset. However, immunocompromised patients may take longer for viral load decrease or have a more erratic viral-load trajectory. We used strand-specific assay data from admitted patients to evaluate viral-load trajectories after illness onset. **Methods:** We reviewed records of hospitalized patients with a positive SARS-CoV-2 PCR and tested using the strand-specific SARS-CoV-2 PCR during July 2020–April 2022. At Stanford Healthcare, we use a 2-step reverse real-time polymerase chain reaction (rRT-PCR) assay specific to the minus strand of the SARS-CoV-2 envelope gene to assess infectivity. Restricting our analysis to each patient’s first strand-specific assay, we used logistic regression models to compare patients with single versus multiple assays. Among patients with multiple tests, we compared those who had an upward trajectory in cycle threshold (Ct) values (a surrogate of decreasing viral load) versus those who did not. We analyzed presence of symptoms, immunocompromised state, immunosuppression reason, and severe COVID-19 leading to ICU care in univariate and multivariate models that further adjust for additional covariates. Significant differences were assessed using logistic regression odds ratios and an α level of 0.05. **Results:** In total, 848 inpatients were included. Among them, 703 were tested only once and 145 were tested 2–6 times. The longest duration of minus-strand detection was 163 days. In univariate analyses, patients with a single minus-strand assay had lower odds of being symptomatic (OR, 0.55), of being immunocompromised (OR, 0.58), and of being admitted to the ICU with severe COVID-19 (OR, 0.49). In the multivariate analysis, being admitted to the ICU with severe COVID-19 was the only significant variable associated with having >1 test (OR, 2.44). Among patients who had multiple strand-specific SARS-CoV-2 assays, 119 had upward minus-strand trends of Ct values (as expected) and 26 did not. Being immunocompromised was associated with nonrising minus-strand CT values (OR, 33.3) when holding all other covariates in the model constant. **Conclusions:** Immunocompromised patients with COVID-19 tend to actively replicate for longer and have unexpected viral trajectories compared to immunocompetent patients. Among immunocompromised patients, suspension of transmission-based precautions may require a case-by-case evaluation.

**Disclosures:** None

